# The role of tobacco and alcohol use in the interaction of social determinants of non-communicable diseases in Nepal: a systems perspective

**DOI:** 10.1186/s12889-020-09446-2

**Published:** 2020-09-07

**Authors:** Sudesh Raj Sharma, Anna Matheson, Danielle Lambrick, James Faulkner, David W. Lounsbury, Abhinav Vaidya, Rachel Page

**Affiliations:** 1 DIYASU Community Development Centre, Biratnagar, Nepal; 2 Massey University, Wellington, New Zealand; 3 Victoria University of Wellington, Wellington, New Zealand; 4 University of Southampton, Southampton, UK; 5 University of Winchester, Winchester, UK; 6 Albert Einstein College of Medicine, New York, USA; 7 Kathmandu Medical College, Kathmandu, Nepal

**Keywords:** Non-communicable diseases, Tobacco, Alcohol, Social determinants, Nepal

## Abstract

**Background:**

Tobacco and alcohol use are major behavioural risks in developing countries like Nepal, which are contributing to a rapid increase in non-communicable diseases (NCDs). This causal relationship is further complicated by the multi-level social determinants such as socio-political context, socio-economic factors and health systems. The systems approach has potential to facilitate understanding of such complex causal mechanisms. The objective of this paper is to describe the role of tobacco and alcohol use in the interaction of social determinants of NCDs in Nepal.

**Method:**

The study adopted a qualitative study design guided by the *Systemic Intervention* methodology. The study involved key informant interviews (*n* = 63) and focus group discussions (*n* = 12) at different levels (national, district and/or community) and was informed by the adapted *Social Determinants of Health Framework*. The data analysis involved case study-based thematic analysis using framework approach and development of causal loop diagrams. The study also involved three sense-making sessions with key stakeholders.

**Results:**

Three key themes and causal loop diagrams emerged from the data analysis. Widespread availability of tobacco and alcohol products contributed to the use and addiction of tobacco and alcohol. Low focus on primary prevention by health systems and political influence of tobacco and alcohol industries were the major contributors to the problem. Gender and socio-economic status of families/communities were identified as key social determinants of tobacco and alcohol use.

**Conclusion:**

Tobacco and alcohol use facilitated interaction of the social determinants of NCDs in the context of Nepal. Socio-economic status of families was both driver and outcome of tobacco and alcohol use. Health system actions to prevent NCDs were delayed mainly due to lack of system insights and commercial influence. A multi-sectoral response led by the health system is urgently needed.

## Background

Tobacco and alcohol use are major behavioural risk factors of non-communicable diseases (NCDs) [1–3]. Worldwide, tobacco and alcohol use are responsible for 8 million NCD-related deaths, mostly in developing countries [3]. These risks are responsible for almost two million NCD-related deaths in the South-East Asia Region (SEARO) of World Health Organization (WHO) alone [4]. SEARO, which has 11 South and South-East Asian countries as its members, including Nepal [5], shares a high burden of tobacco users with 20% of smokers and 80% of smokeless tobacco users [6]. In the case of alcohol consumption, while the global per capita consumption of alcohol is low in the region, SEARO has recorded a significant increase in the per capita consumption (2.2 l in 2005 to 3.4 in 2010) and accordingly, an increase in the prevalence of current drinkers (10.7 in 2005 to 13.5 in 2010) [4]. In Nepal, about 27,000 people die every year from tobacco-related deaths while alcohol is responsible for about 6500 deaths every year [7]. Although Nepal has strong tobacco and alcohol control policies, the evidence consistently shows high prevalence of tobacco and alcohol use. The STEPwise approach to chronic disease risk factor surveillance (STEPS) survey in 2014 indicated that 31 and 17% of adults are current users of tobacco and alcohol products respectively [1]. With such high prevalence of tobacco and alcohol use in Nepal, it can be expected that NCD-related mortality will continue to rise.

WHO has identified the prevention and control of tobacco and alcohol use as key strategies to prevent NCDs and resulting deaths [3]. In recent years, there have been active efforts by global health agencies and experts to move beyond prevention of these immediate behavioural determinants and towards addressing social determinants of health and NCDs [8, 9]. This shift to address social determinants has been rapid in developed countries but much slower in developing countries like Nepal. Developed countries like Australia and New Zealand are leading in their efforts to control tobacco and alcohol use among their disadvantaged (particularly indigenous and low-income population) groups by taking action on the social determinants of tobacco and alcohol use [10–13]. These countries have gradually reduced inequities in health status among population sub-groups through integrated social, economic and health policies and programs. In developing countries like Nepal, there is a significant gap in understanding and addressing these social determinants of tobacco and alcohol use and inequities in health status. Some local evidence from Nepal does show poverty, illiteracy, and low-skilled occupations significantly associated with tobacco and alcohol use [1, 14]. However, tobacco and harmful alcohol use prevention policies and programmes in developing countries continue to ignore the social determinants side of tobacco and alcohol use. Commercial influence and its impact have been noted globally [15] but not locally. The ability of the health system to address the social determinants of tobacco and alcohol use and NCDs is yet to be focused. Understanding how poverty, socio-economic situation, industrial influences and health system determinants interact with tobacco and alcohol problem and the unfolding burden of NCDs is paramount in the context of Nepal. The objective of this paper is to describe the role of tobacco and alcohol use in the interaction of social determinants of NCDs in Nepal.

## Method

**Fig. 1:**
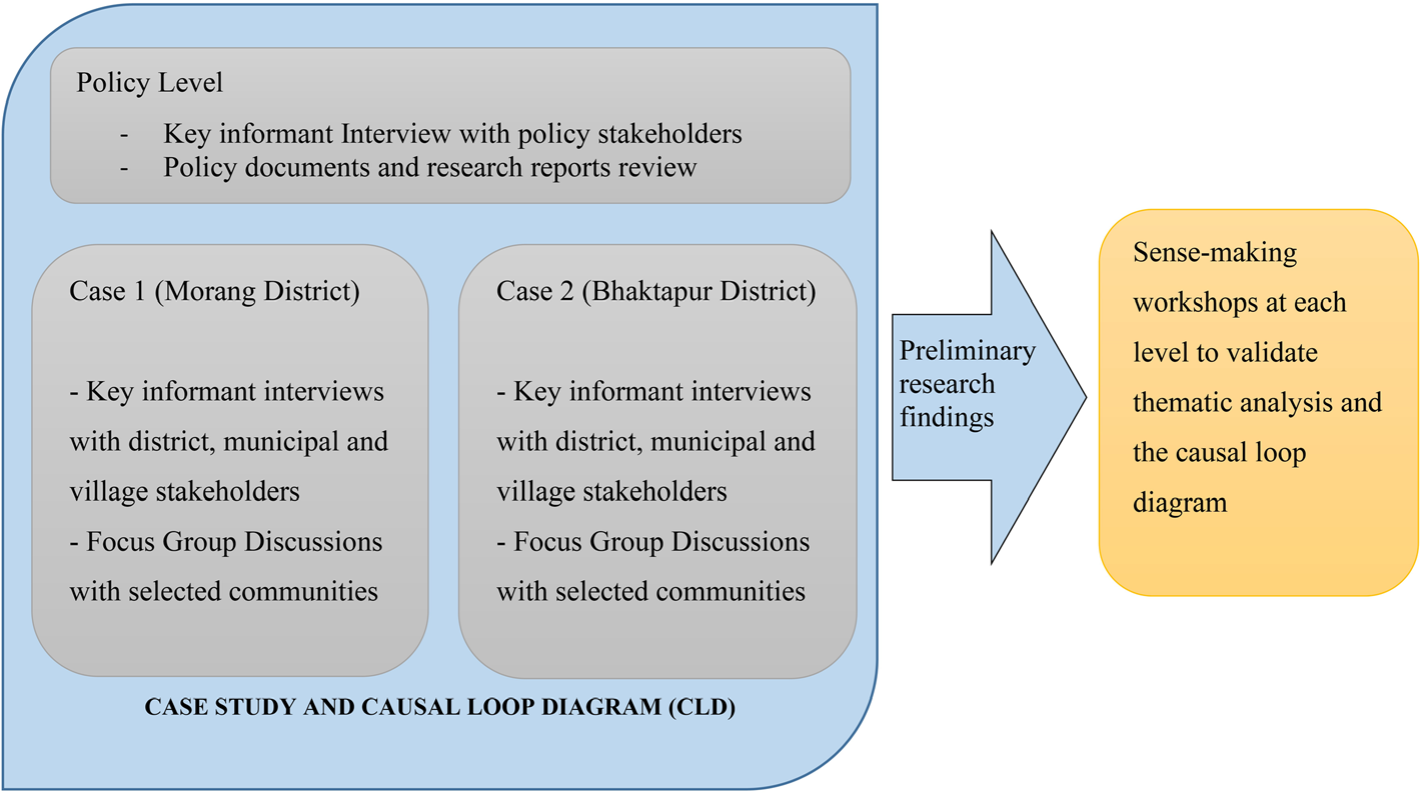
Systemic Intervention design of the study of social determinants of NCDs in Nepal

The study adopted a qualitative study design (Fig. 1) and informed by a systems science methodology, namely systemic intervention (SI) [16]. Systems science is being increasingly applied to understand and tackle multi-level, complex problems in population health [17, 18]. Systems science helps to understand how complex problems are emergent and generated from the dynamic interaction of multiple parts and facilitates richer understanding and continuous learning [19–21]. As such, systems methods and tools are well suited for illuminating the dynamic structure and emergent behaviour of complex health problems and their social determinants. While systems science approaches have been increasingly utilised for understanding and modelling complex public health issues in developed countries [18, 22–24], there are far fewer instances of their application in developing country contexts [25, 26]. In particular, SI takes a critical systems approach while promoting use of multiple methods from different disciplines. In this study, the critical systems approach has resulted in the meaningful representation of diverse and marginalised groups most affected by NCDs. A combination of two methods was applied: a case study approach [27] and system dynamics [21]. The case study method framed the scope of the qualitative data collection and analysis to understand the generative mechanism of the NCDs, particularly the influence of context. A system dynamics method was used to design causal loop diagrams (CLDs), which depicted the relationships and interactions identified through case study analysis.

### Case study approach

#### Study area and participants

Two geographically defined districts were purposively selected as cases for this research. These districts were Morang district from Terai (plain) region, and Bhaktapur district from Hill region. Each case study involved key informant (KI) interviews with national, district and village level stakeholders, and focus groups (FGs) with community people. The district and Village Development Committees (VDCs) /municipality level KIs were identified through consultation with District Public Health Offices and included participants from District Health Office, Local Development Office, local non-government organisations (NGOs), Primary Health Centres, health posts, local schools and Village Development Office. One municipality and two VDCs from each case district were selected for interviewing key local stakeholders.

The District Public Health Offices helped to identify two communities (one advantaged and other disadvantaged communities) within each VDC /Municipality for FGs. Local health workers and Female Community Health Volunteers (FCHVs) supported the first author in the planning and conducting FGs in the target communities. National level KI interviews were conducted to expand the perspectives of the case studies and comprised of multi-sector participants involved in formulating the *Multi-sectoral Action Plan for the Prevention and Control of NCDs 2015*–*2020* and from NGOs and academia. The participants recruited for KI interview varied in terms of workplace, years of experience, sectors (health as well as non-health), and expertise (implementation as well as national level). The study adopted a “maximum variation” sampling strategy to collect perspectives on NCD issues from across the sectors [28].

#### Study tools

**Fig. 2:**
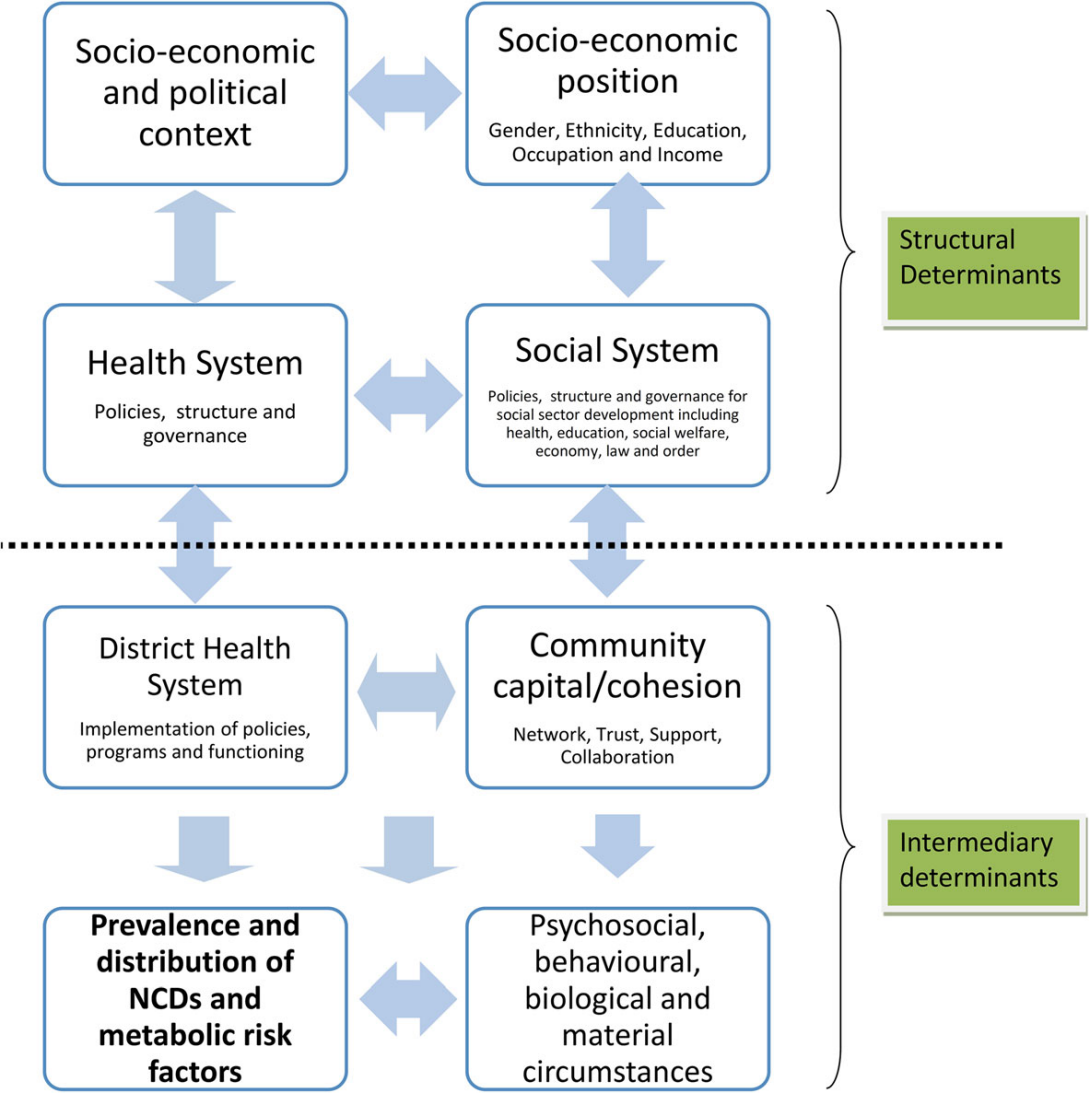
Study framework adapted from the *Social Determinants of Health Framework* of World Health Organization

The study tools (KI interview schedule and FG guidelines were informed by the study framework adapted from the social determinants of health (SDH) framework of the World Health Organization (Fig. 2) [See Additional file 1]. The study tools were extensively discussed in light of the adapted framework by the research team and they were refined following the first round of interviews. The tools were first developed in English and translated into Nepali.

#### Data collection and analysis

The data collection was undertaken over four months (July–October 2016) in Nepal. Formal ethical approvals were obtained from the Massey University Human Ethics Committee (SOA 16/37) and Nepal Health Research Council Ethics Committee (Reg. no. 163/2016) respectively. A prior informed and written consent was obtained from all participants for KI interviews and FGs.

##### KI interviews

The first author interviewed 39 KIs from the two case districts and 24 KIs from national level. The time of interview ranged from 30 min to one hour. The first author simultaneously started the district and community level data collection at Bhaktapur district and at national level. The research team utilised the framework approach to code the qualitative data and carry out the thematic analysis guided by the study framework (Fig. 2) [29]. Interviews were audio-recorded, transcribed in Nepali and then translated into English. The translations were carried out by two public health graduates from Nepal and were regularly supervised by the first author. Open descriptive coding, guided by the study framework, was done by the first author in *Dedoose*, a web-based data management platform [30]. The first author coded a few interviews first and compared the transcripts for consistency and clarity in coding. The final codes were then grouped and charted in *Ms Excel* sheet and key themes were developed based on the study framework. Causal linkages among social determinants were interpreted and mapped from the key themes.

##### Focus groups

The first author conducted 12 FGs in six selected VDCs/municipalities from the two case districts involving five to 10 community participants affected by and/or caring for family members with NCDs metabolic risks. Half of the FGs (one per VDCs and municipalities) were conducted in socio-economically disadvantaged communities identified through the help of local health workers. The time of an FG ranged from 45 min to one hour. The process of transcription, translation and analysis was similar to the KI interviews.

### The causal loop diagram

The causal relationships and interactions identified through two cases were depicted together in the form of CLDs. CLDs are a qualitative approach used in system dynamics modelling to identify feedback loops and structures that illustrate causal influences for a given problem of interest [21].. CLDs comprise two kinds of loops: balancing and reinforcing. The balancing loop is a goal-seeking loop, which is indicated by “B” within CLD and indicates a stabilising feature of the loop. Generally, loops encompassing health intervention actions (health education campaign, screening, treatment, etc), which aim to bring down the magnitude of health problems, are examples of balancing loops. Reinforcing loops, on the other hand (indicated by “R” within CLD), involve action that may produce a result that triggers actions that reinforce the current system trajectory; for example, the vicious cycle of poverty and illness. The CLDs are often complex, so a simpler version of CLDs called system archetypes was developed to understand the complex causal mechanism. System archetypes are simple templates of CLDs for understanding common problems or dilemmas in an organisation or system and in a way that generates insights for action [31, 32]. The CLDs and archetypes were built using the *Vensim* software [33].

### Stakeholder validation

Stakeholder validation involved organising three workshops, two within the case districts and one national level workshop. These were carried out during January/February, 2018. The first author provided information about the research including description of the systems thinking approach and CLD. The author then presented the seed CLD structure, corresponding themes and direct quotes and added more social determinants variables (and causal linkages) to the seed CLD structure showing the interaction among different social determinants variables. Participants were encouraged to present their views and question anytime during the presentation. These workshops helped to further improve the CLDs and the qualitative analysis through the feedback and suggestions from the stakeholders. The workshops also served as an opportunity to share knowledge about the adverse consequences of the tobacco and alcohol practices in Nepal.

## Results

Three key themes and CLDs relating to social determinants of tobacco and alcohol were derived from the analysis which are as follows:

### Theme 1: exposure and availability of tobacco and alcohol products

According to KI and FG participants, awareness of the impacts on health of tobacco and alcohol use was widely present among the general population in both rural and urban areas. However, despite this knowledge about risks of smoking and drinking, it was reported that people continue to indulge in these risky behaviours. Participants reported that adults from the study areas were often exposed and thus become addicted to these products at a younger age. Young people have easy access to products from liquor and tobacco shops despite being under the legal purchasing age. A health worker from urban Bhaktapur stated:

*“8*–*9 class students smoke tobacco who can get them easily from the shops.” (ID: 42)*

Some participants reflected that the use of tobacco and alcohol was also driven by misconceptions. One common misconception was that tobacco and alcohol offered the user relaxation and reduced physical and mental stress. An FG participant candidly shared:

*“Not only smoking alleviates tiredness, if one smokes, then one gets some rest from work.” (ID: 74)*

KIs and FG participants suggested that community capital and cohesion were declining, which was contributing to limited community action by concerned citizens. When potentially effective actions were initiated by communities, especially those by women’s groups, for example to reduce alcohol abuse, they were often short-lived due to the lack of support from male members of the community and community leaders.

*“We have tried to address this many times. But whenever women raise their voice against these, pub and shop owner quarrel with them. Police was sought for help but they didn’t take any action.” (ID: 56; Village level KI; Rural Bhaktapur)*

Local shop owners within these same communities often diversify their sales to include the supply of alcohol, as this can help to supplement their income when they themselves are facing economic hardship. An FG participant from rural Morang stated:

*“They (shopkeepers) say they won’t make money if they do not sell alcohol.” (ID: 68)*

KIs suggested that local shop owners frequently put personal economic benefits before social and health consequences, and were selling products without conscience, even to underage groups. A social worker from urban Bhaktapur shared:

*“And why would business people think before selling; those college students are the source of profit. Profit margin is high in alcohol and cigarettes. Ethics and values are neglected by such business owners.” (ID: 44)*

It was reported that home-made alcohol producers sometimes used hazardous chemicals and toxic substances to amplify alcohol strength as a means of attracting more customers.

*“What I have heard is that they use inedible substances including animal remains. They try to make strong alcohol using urea fertilizer. That can severely affect our health.” (ID: 76; FG participant; Rural Bhaktapur)*

Alcohol and tobacco were not considered a significant problem by local authorities. This was illustrated when one of the district level KIs from Morang shared that concerns about tobacco and alcohol use never entered the local planning agenda.

*“Due to this, during planning process from the community level (planning must start from the community level) the issues regarding the prohibition of alcohol and tobacco products etc. aren’t arisen while discussing about the plans.” (ID: 50)*

### Theme 2: limited focus on primary prevention of tobacco and alcohol use by the health system

It was clear through the interviews that there has been a lack of focus on primary prevention of NCDs, tobacco and alcohol use and their social determinants at the national level. A curative orientation - focusing on treatment or cure of a health problem rather than preventing the occurrence of the problem -was clearly dominant at both national and implementation levels of health sector. Revenue raised through tobacco and alcohol taxes was more often used for curative and other non-health budgetary purposes. Very rarely, if ever, would these resources be used for preventing tobacco and alcohol use through multi-sectoral approaches or tackling commercial influences. A national level stakeholder, with experience of working in tobacco and alcohol prevention program, shared,

*“Finance Ministry do not provide enough resources (for primary prevention) despite huge amount is generated from (excise) tax.” (ID: 14; National level KI)*

Participants argued that weak monitoring and enforcement of regulations were leading to unabated production, marketing and availability of tobacco and alcohol products.

*“Implementation of tobacco control policies is not effective at all. Is 500 meters no sale near school effective? It cannot be possible under current system.” (ID: 35; Village level KI; Bhaktapur)*

Participants indicated that tobacco and alcohol industries are the major source of revenue and have strong linkage with policy makers. They have been influencing policy decisions in their favour. A national level stakeholder shared one of his experiences as follows:

*“Tobacco production companies filed a case in Prime Minister’s office and the Prime Minister directed officials not to change the existing rule till the [proposed Tobacco Control Law] law was passed and to reconsider the practicality to change [pictorial warning image] from 75% to 90% within the law and take decision accordingly.” (ID: 23; National level KI)*

Participants at both national and district level also expressed that the district and community health system did not have any well-resourced programmes for preventing tobacco and alcohol use. Neither were there any counselling support services for those already addicted to tobacco and alcohol.

*“These tobacco, tobacco products and drugs become addiction to people. We apply the prevention approach to those who don’t consume these substances. For those who consume these substances, rehabilitation and counselling must be strengthened.” (ID: 50; District level KI; Morang)*

Most of the national level KIs described a lack of a focused policy structure and leadership for initiating any multi-sectoral action for the primary prevention of NCDs. According to one national level stakeholder, a division within the Ministry of Health responsible for curative services by hospitals around the country was leading the multi-sectoral action, which indicated gaps in the policy structure and function for NCDs prevention. The national level stakeholder shared:

*“Curative Service Division is leading this fight against NCD but more from curative perspective and less from Health promotion.” (ID: 15)*

Even where resources have been explicitly allocated to NCD prevention, KIs argued that their use has been ineffective because of fragmentation and misallocation.

*“There is budget for NCD prevention but they are scattered in various places. That has to be managed through certain centre in an effective way.” (ID: 12)*

### Theme 3: gender and socio-economic status as the root drivers of tobacco and alcohol use

Gender and socio-economic status have been identified as key drivers of tobacco and alcohol use. From a gender perspective, participants reported that tobacco and alcohol use were implicitly driven by gendered social constructs and the way in which power relationships played out. Study participants shared that it was mainly men within their communities that demonstrated addictive behaviours. They further suggested that this situation of widespread addiction among men could be linked to a combination of factors, including the need to relieve stress, financial autonomy, and the perceived lower social status of females.

*“Male are more intensely involved in alcoholism. They earn money during day time and spend it on drinks at night. This problem is more intense among 6–7 household in our locality. Even domestic violence is common in those houses.” (ID: 56; Village level KI; Morang; Health)*

One FG participant from rural Morang was vocal about the increased stress on women due to the drinking habits of men, and their inability to do anything to address it.

*“You males drink, smoke and this problem [hypertension and diabetes] is because we take stress about that.” (ID: 67; Female FG Participant; Rural Morang)*

Some KIs also noted there is a recent, increasing trend in tobacco and alcohol use among females, with one national level informant suggesting that there might be an underestimation of female tobacco and alcohol use within national surveys. This may be due to the social pressure on women to not be seen as consumers of these products.

*“There is the perception in our society that females shouldn’t be consuming such substances and so females do not give true answers and also our enumerators may not have been able to explore effectively.” (ID: 5)*

Participants shared that tobacco and alcohol use were a major community problem among low-income groups. Tobacco and alcohol were seen as a way to ward off the stresses of daily life.

*“Most of the people here are engaged in labour work. They have to do hard work like carrying stones and get tired and do not even eat their food on time. In the evening to get rid of their tiredness, they consume alcohol.” (ID: 37; Village level KI; Bhaktapur)*

A community level KI reported that those from low-income communities operated many shops selling alcohol and tobacco. Many of these businesses have been borne out of a need to earn money amidst a dire lack of job opportunities.

*“However, these home-made alcohols are the means to earn money for the small shops and poorer households.” (ID: 55; Village level KI; Morang)*

There is evidence of diminishing boundaries between traditional drinkers (Gurung, Rai, Magar, Newar and similar ethnicities – collectively referred as *Matwali* – who are culturally allowed to drink alcohol) and traditional non-drinkers (Brahmin, Chhetri and similar ethnicities – collectively referred as *Tangadhari* – who are culturally forbidden to drink alcohol). This has led to increased total alcohol consumption within the case districts, especially among those who are poor, irrespective of ethnicity.

*“There was social rule that it is something to be consumed by Matwali but not by Brahmins and Chhetris but now the situation has just reversed. These days it is hard to find Brahmin/ Chhetris who do not drink.” (ID: 47; District level KI; Morang)*

Alcohol is very much ingrained in the cultural practices of the *Matwali* ethnic group. Many of their rituals and cultural practices involve alcohol. Due to poor socio-economic status, home-brewing in *Matwali* communities is commonplace and these products are being supplied to shops around the locality as well as nearby cities. As such, *Matwali* have begun to use their traditional skills for home-brewing to produce on a commercial scale due to the monetary incentive. A national level stakeholder explained the situation:

*“Matwali have cultural practice of brewing home-made alcohol and we do not infringe into that cultural practices. But, many have been exploiting this cultural aspect for economic benefits including those who were non-traditional brewers.” (ID: 16)*

### The causal loop diagram (CLD): interactions of tobacco and alcohol use and NCDs

The key themes were utilised to interpret and map possible interactions among the social determinants. Three interacting CLDs or sub-systems and corresponding system archetypes were generated. These interacting sub-systems displayed some key sets of balancing and reinforcing loops that are possibly escalating the NCDs epidemic in the context of Nepal.

#### Demand-supply sub-system

**Fig. 3:**
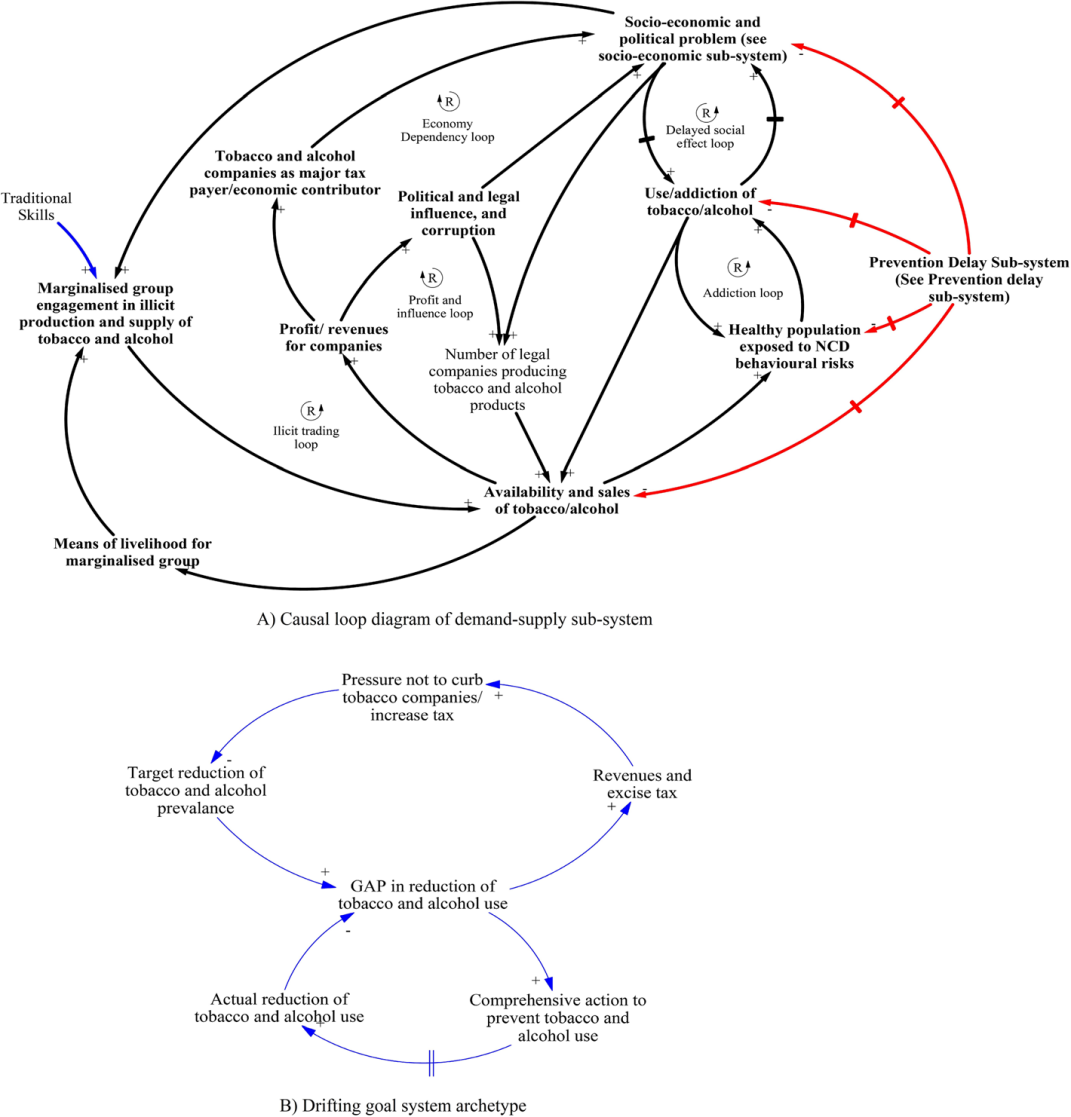
Causal Loop Diagram (CLD) of demand supply sub-system and drifting goal archetype

As presented in the first theme, this sub-system illustrates that tobacco and alcohol use were being reinforced by the widespread availability and sales of such products in the case districts of Nepal (Fig. 3). Industries that produce tobacco and alcohol have financial capacity for marketing to vulnerable groups as well as influence in policy decisions (such as delaying rapid increase in excise tax) in their favour as illustrated by profit and influence loop and *drifting goal* archetype in Fig. 3. Another key reinforcing loop was the illicit trading loop, which illustrated the role of marginalised or disadvantaged groups in the sales of home-made alcohol and tobacco products.

#### Prevention delay sub-system

**Fig. 4:**
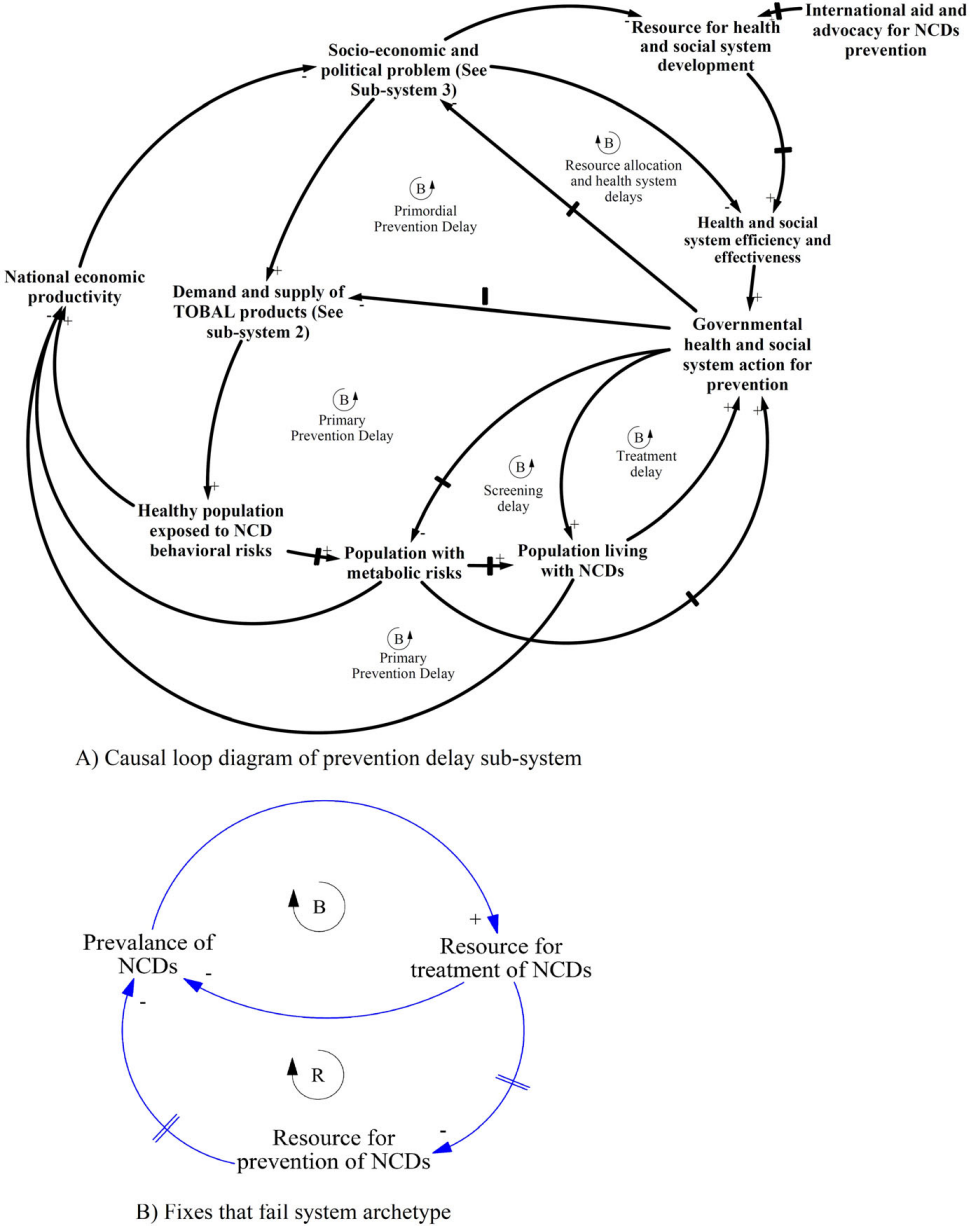
Causal loop Diagram (CLD) of prevention delay sub-system and fixes that fail system archetype

The delays in primary prevention and multi-sectoral actions have been illustrated by delayed balancing or intervention loops (Fig. 4) as discussed in the second theme. The negative sign between “Government health system action” and “demand and supply” here means that increasing implementation of regulations and monitoring can decrease availability. However, in this circumstance the action is delayed (indicated by a delay sign in the arrow i.e. //), resulting in increasing exposure of the healthy population to tobacco and alcohol products, which leads to metabolic risks and NCDs (links have positive sign). The prevention delay sub-system resonates with *Fixes that fail* systems archetypes, indicating a failed strategy of allocating more resources towards the treatment of NCDs rather than for prevention through multi-sectoral effort.

#### Socio-economic influence sub-system

**Fig. 5:**
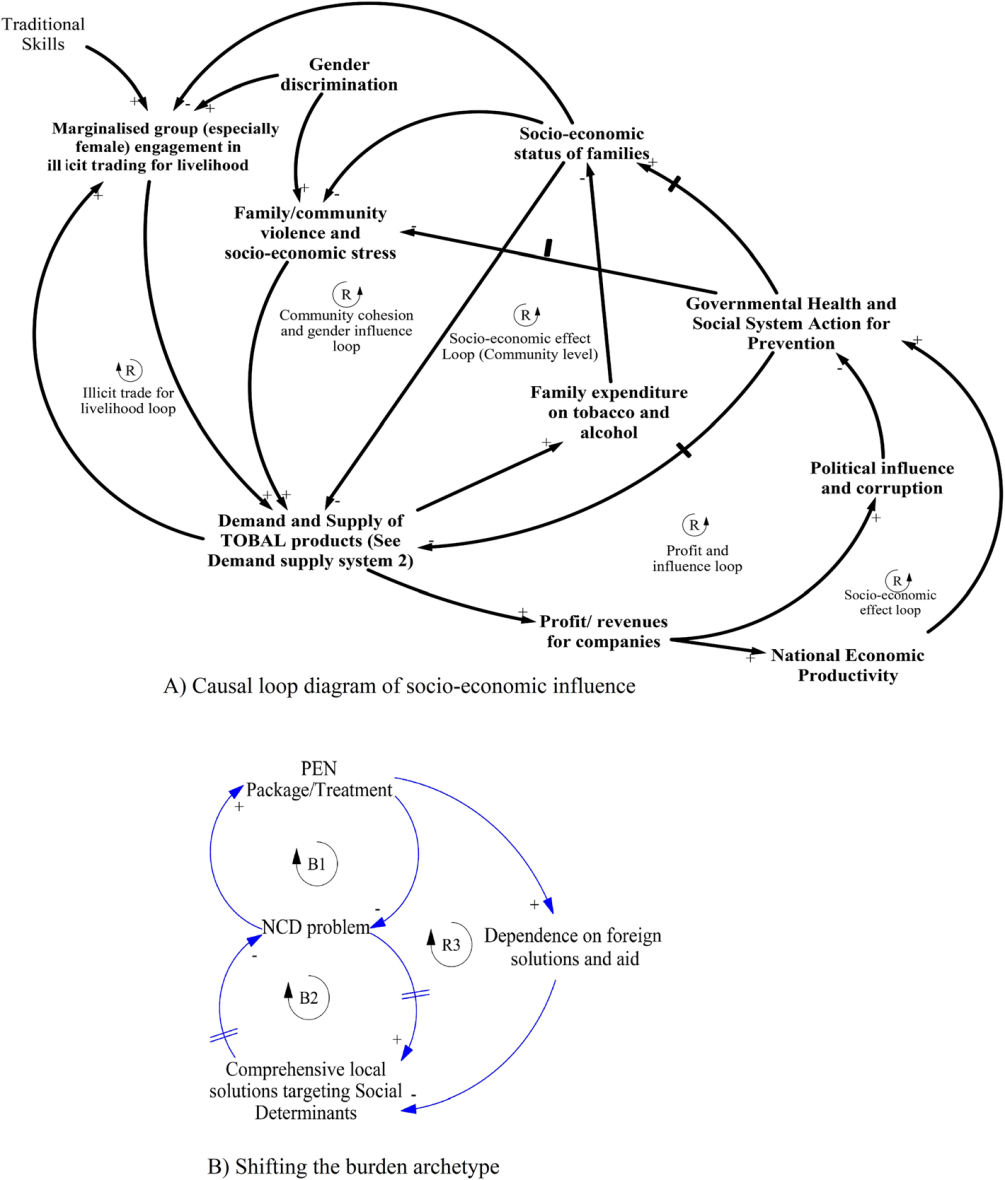
Causal Loop Diagram (CLD) of socio-economic status influence and shifting the burden system archetype

This sub-system contains reinforcing loops, which illustrate the social and economic influences contributing to the current environment for tobacco and alcohol (Fig. 5) based on the third theme. In particular, a reinforcing mechanism of the socio-economic hardship leading to stress, gender-based violence and misconceptions, and eventually to tobacco and alcohol use and addiction is shown. Further, socio-economic hardship among specific disadvantaged groups, for example *Matwali*, meant that the socio-economic status was reinforcing the supply of home-made alcohol through illicit trading. *Shifting the burden* archetype (Fig. 5b) depicts the inability of the health system to see the bigger picture or broader influences driving the NCDs problem. This demonstrates that the Nepalese health system has been focusing on narrow sets of interventions driven by foreign support and ignoring the complexity of the issue, which is embedded in the socio-cultural context and therefore demands a more local solution.

## Discussion

The three themes and CLDs (sub-systems and system-archetypes) have helped to illustrate the dynamics of the interaction between the social determinants of NCDs and tobacco and alcohol use in Nepal. In this study, tobacco and alcohol use were common, particularly among the disadvantaged groups. Some of the use was driven by a popular misconception that tobacco and alcohol relieve stress. This reasoning has been found to be prevalent among low-income populations in both developed and developing countries [34–37]. The evidence is clear that smoking in fact increases stress in part as a result of cravings [38, 39]. Widespread use of tobacco and alcohol products were facilitated by many factors including the social and cultural acceptability of such products in Nepalese society [40, 41]. However, key reasons for the wider use of such products was mainly due to easy availability of such products, commercial influences in policy and delays in administering widespread and effective preventive strategies and policies. Tobacco and alcohol industries have been successful in undermining public health policies and actions in Nepal and exacerbate the high prevalence of tobacco and alcohol use, especially among vulnerable groups. For example, tobacco industries have been specifically targeting young people from developing countries [42, 43]. Targeting youth has two main benefits for these industries: youth may be more easily influenced, and once they start using these products, they are likely to use them for a longer duration. Further, the influence of tobacco and alcohol industries is such that tobacco and alcohol control policies are often poorly resourced and implemented, without real commitment by the overall government system (and in particular, the health system). In developing countries like Nepal, tobacco and alcohol industries have successfully argued that they contribute significantly to national income and have fostered and maintained favourable relationships with national level policy makers [43]. As suggested in the *Drifting goal* archetype, policy makers in Nepal are not willing to raise excise tax on tobacco products as per the international standard [44], likely due to the political connections and influences of the tobacco and alcohol industries at national and local levels.

The prevention delay subsystem and *fixes that fail* archetype show that the health system response has been delayed and ineffective in addressing the social determinants of tobacco and alcohol use. Despite Nepal being among those countries with comprehensive tobacco and alcohol control laws and policies, limited resources for prevention, including regulatory action, have resulted in increased availability of tobacco and alcohol products. Similarly, a high prevalence of tobacco use has been noted in many developing countries where similar laws exist but are poorly implemented [45–47]. Tobacco and alcohol industries are increasingly focused on developing countries where system mechanisms are weak and can be bought and influenced [15, 48]. Inefficiency, poor governance and lack of leadership within health and social systems have been cited as the main system issues exacerbating complex problems like tobacco and alcohol use in developing countries [49–51]. In contrast, developed countries have begun to align their health system actions in order to address complex and shared public health problems [52, 53]. Over time, they have been able to implement effective tobacco control policies and reduce the prevalence significantly [54].

South Asian countries are patriarchal societies, with men enjoying more power and autonomy and engaging in more risky behaviour compared to women [14, 55, 56]. The gender power gap and disproportionate levels of smoking and drinking among males have put females from low-income groups at a significantly higher risk of gender-based violence in the case districts. The impact of addictive behaviour in terms of violence and socio-economic stress on women and children has been noted globally, such as in Cambodia, India and Bangladesh [57–61]. A study in China indicated that women accepted the addictive behaviour of their husbands to maintain family harmony, illustrating the sub-ordinate and low status of women within the family [62]. A study in India noted that women who experienced domestic violence eventually started tobacco use, which illustrated one of many effects of gender-based violence [63]. Interestingly, there is some evidence to suggest that women tobacco and alcohol users could be rising in developing countries due to gender empowerment, a loosening of socio-economic constraints and targeted campaign by such industries [64]. Although the underlying reasons have not been explored in our study, we have shown evidence that alcohol and tobacco use may be underreported in women in Nepal, which limits our current understanding of the true scale of the issue.

Tobacco and alcohol use often lead to huge economic losses [65, 66] and push individuals and families into a vicious poverty cycle. Similar to the findings of this study, studies have shown that children and youth from disadvantaged communities are often exposed to alcohol use at very early age [41, 67]. Often, these children gradually drop out of school and add to a non-skilled workforce with addictive behaviours and poor health. One prospective study from the United States suggested similar socio-economic and health impact of alcohol on adults who were exposed to alcohol at an early age [68]. While there was supposedly a high prevalence of home-made alcohol abuse in the traditional drinking ethnic group (*Matwali*), these groups have been historically marginalised and often have poor socio-economic status [69]. Further, there appeared to be a rapid increase in alcohol consumption in the traditional non-drinking ethnic group (*Tangadhari*), especially among low socio-economic groups. This shift has been noted in research carried out almost two decades ago in Nepal [41]. This indicated that the use and addiction of tobacco and alcohol products were being mainly influenced by socio-economic status rather than ethnicity.

In this study, small businesses within communities sold tobacco and alcohol widely and communities did not offer any resistance against widespread availability of such products. This community inaction was linked to dynamic interaction of community capital, gender and socio-economic situation. The findings indicated that social capital was declining and hampering collective action. Increasingly, studies have shown the relationship between community capital, collective action and health outcomes, and therefore the case districts were missing out on leveraging social capital for preventing tobacco and alcohol use locally [70, 71]. Furthermore, women, both as individuals and as groups, had limited ability to take collective action against the availability of such products and use by their male counterparts due to their low social status as discussed above. In this study, disadvantaged families have been utilising their traditional skills to produce and sell alcohol to overcome their financial situations in both case districts. Evidence indicated that most of the small businesses operated by people from low-income households sold tobacco and alcohol products in order to make ends meet [14, 72]. As a result, other sections of communities had little power and agency to counter this local availability. Any action against the disadvantaged group raises social and ethical dilemmas about taking away their livelihoods without also providing an alternative means of generating income.

There were some key limitations of the study. First, the study design and tools were guided by the WHO SDH Framework and hence may have been affected by the limitations that are inherent to the SDH Framework itself, including being broad and wider in scope. Secondly, some of the determinants that could not be sufficiently supported by the data included financial burdens and their implications on the families affected by tobacco and alcohol as well as lived experience of the people with tobacco and alcohol addiction and NCDs. Future studies that focus on the lived experience and on a few key determinants may help to further elucidate the acceleratory effects of tobacco and alcohol use on the NCD epidemic in Nepal. There were some methodological limitations as well. The participants of the workshops were mainly from the health sector. This may have weakened the feedback process where we expected feedback from multi-sector participants. The CLDs also mainly represent the mental model of the authors based on the thematic analysis and hence, should be interpreted carefully. However, the approach within the study does present an opportunity to further engage key stakeholders in transforming insights from current CLDs into collective action and learning [73].

## Conclusion

The research findings could be utilised in two ways: i) to broaden one’s understanding of the role of tobacco and alcohol use in the interaction of the SDH, and ii) to identify systemic actions for addressing such complex challenges from a systems perspective in Nepal and similar developing countries. This research illustrates how addiction and product availability were influenced by wider socio-economic determinants, and how the health system in Nepal is failing to tackle NCDs from an SDH perspective. Socio-economic status of families not only pushed people into the habit of tobacco and alcohol use but also exposed females and children to domestic violence and perpetuated the vicious cycle of addiction and poverty. The sub-systems and archetypes informed by the current case study districts are a starting point for critical dialogue and action in Nepal in understanding and addressing the complex issue of reducing behavioural risks and in mitigating the burden of NCDs. The balancing effects of a health system to prevent NCDs have already been significantly delayed, leading to an accumulation of NCD burdens. Two key systemic action for health system of Nepal to impact the accumulation of NCDs include reorienting health system from curative focus to primary prevention of NCDs and behavioural risks, and leading the multi-sectoral action in addressing the social and commercial determinants that are driving the use of tobacco and alcohol.

## Supplementary information

**Additional file 1.** Study tools.

## Data Availability

Transcripts (without any personal identifier) and study tools are available on request (Email: yoursudesh@gmail.com; r.a.page@massey.ac.nz). This paper is part of the PhD study of the first author, and after completion of the PhD study, all transcripts will be available through an open access data repository.

## Abbreviations

CLD: Causal Loop Diagram
FG: Focused Group
KI: Key Informant
NCDs: Non-communicable diseases
SDH: Social determinants of health
SEARO: South-East Asia Region
STEPS: STEPwise approach to chronic disease risk factor surveillance
VDC: Village Development Committee
WHO: World Health Organization
