# Supplementary material for: The role of tobacco and alcohol use in the interaction of social determinants of non-communicable diseases in Nepal: a systems perspective
Source: BMC Public Health. 2020 Sep 7;20:1368. doi: 10.1186/s12889-020-09446-2 (PMC7487957; doi:10.1186/s12889-020-09446-2)
Supplement: Supplementary file 1 — Additional file 1. Study tools. [file 12889_2020_9446_MOESM1_ESM.docx]

**Additional file 1: Study tools**

***Key informant interview with Policy level informants***

Date:

Organisation:

- Can you please share your brief background and current role in your organisation?
- How do you consider the current situation of NCD in Nepal?
- How are you/your organisation associated with prevention of NCD or its risk factors or both?
- What are the key challenges or barriers in the primary prevention and control of NCD at policy and implementation level?

Policy level

- - NCD specific policies and programs formulation and implementation
  - Laws formulation and implementation
  - Funding for primary prevention of NCD
  - Multisector policies and collaboration mechanism
  - Health System structure and functioning
  - Governance (corruption and political influence)
  - Socio-political and economic context including poverty, development and federalism
  - Global context including trade policies and globalisation

Implementation level

- - Individual behaviours
    - Risk behaviours such as tobacco use, alcohol consumption, salt intake, diet, physical activity: prevalence, age group affected, reasons for risky behaviours, cultural factors
  - District (Public) Health Office Functioning/Skills of health workers
  - Female Community Health Volunteers mobilisation
  - Multisectoral collaboration
  - Local socio-economic, political and cultural factors (including community capital/stakeholder collaboration)
- What could be the way forward in the primary prevention and control of NCD at policy and implementation level?
- Is there anything else that you would like to tell us so that we have a better understanding of the NCD situation in the country, or are there any issues you feel are important that we have not touched on during the interview?

At the end of the interview:

- Thank the interviewee for taking time to share the valuable information

***Key informant interview with District level stakeholders***

Date:

Organisation:

- Can you please share your brief background and current role in your organisation?
- How do you consider the current situation of NCD in Nepal?
- How are you/your organisation associated with prevention of NCD or its risk factors or both?
- What are the key challenges or barriers in the prevention and control of NCD at district level?
  - Policy and directives from centre
    - Policy and programs for NCD prevention and control in district
    - Interaction with policy personnel in NCD matters
  - Individual behaviours
    - Risk behaviours such as tobacco use, alcohol consumption, salt intake, diet, physical activity: prevalence, age group affected, reasons for risky behaviours, cultural factors
  - District Health Office Functioning
    - NCD prevention programs and services including FCHV Services and PHC ORC
    - Skills of Human Resources
    - Medicines and Technologies
    - Recording and reporting
    - Multisectoral collaboration
  - Multisectoral actions if any (health, food, occupation, education, social development, law) including District Development Council planning and budgeting
  - Socio-economic, political and cultural factors (including community capital/stakeholder collaboration)
    - Local socio-economic and political context
    - Poverty, Ethnicity, Occupation, Education situation
    - Community capital (Neighbourhood, Social inclusion, Social Groups) and community participation in health facilities and services
- What could be the way forward in the prevention and control of NCD at district?
- Is there anything else that you would like to tell us so that we have a better understanding of the NCD situation in the district, or are there any issues you feel are important that we have not touched on during the interview?

At the end of the interview:

- Thank the interviewee for taking time to share the valuable information

***Key informant interview with VDC/Municipality stakeholder***

Date:

Organisation:

- Can you please share your brief background and current role in your organisation?
- How do you consider the current situation of NCD?
- How are you/your organisation associated with prevention of NCD or its risk factors or both?
- What are the key challenges or barriers in the prevention and control of NCD at community level?
  - Individual behaviours
    - Risk behaviours such as tobacco use, alcohol consumption, salt intake, diet, physical activity: prevalence, age group affected, reasons for risky behaviours, cultural factors
  - Health institution functioning and support
    - NCD prevention programs and services including FCHV Services and PHC ORC
    - Skills of Human Resources
    - Medicines and Technologies
    - Recording and reporting
    - Multisectoral collaboration
  - Multisectoral actions if any (health, food, occupation, education, social development, law) including VDC/Municipality planning and budgeting
  - Socio-economic, political and cultural factors (including community capital/stakeholder collaboration)
    - Local socio-economic and political context
    - Poverty, Ethnicity, Occupation, Education situation
    - Community capital (Neighbourhood, Social inclusion, Social Groups) and community participation in health facilities and services
- What could be the way forward in the prevention and control of NCD at community level?
- Is there anything else that you would like to tell us so that we have a better understanding of the NCD situation in the community, or are there any issues you feel are important that we have not touched on during the interview?

At the end of the interview:

- Thank the interviewee for taking time to share the valuable information

***Focus Group Study Guidelines***

- Can you please share your brief background and the metabolic risk factor (High BP, Blood sugar or BMI) of NCD you (or family member) are suffering from?
- How do you consider the current situation of NCD in your community?
- What are the key challenges or barriers in the prevention and control of NCD at community level?
  - Individual behaviours
    - Risk behaviours such as tobacco use, alcohol consumption, salt intake, diet, physical activity: prevalence, age group affected, reasons for risky behaviours, cultural factors
  - Material circumstances
    - Communities and neighbourhood environment like violence, poverty level, education level and housing status, parks and recreations, roads, public transport
    - Material availability and adequacy for quality of life and wellbeing (housing, water, sanitation, fuel wood, shops)
  - Psychosocial factors
    - Individual Stress especially work and family related
    - Social stress relating to living conditions and social cohesion
  - Community capital/Cohesion
    - Network, Trust, Support, Collaboration
  - Health institution functioning and support
    - NCD prevention programs and services including FCHV Services and PHC ORC
    - Skills of Human Resources
    - Medicines and Technologies
    - Multisectoral actions if any (health, food, occupation, education, social development, law) including VDC/Municipality planning and budgeting
  - Socio-economic, political and cultural factors (including community capital/stakeholder collaboration)
    - Local political context
    - Poverty, Ethnicity, Occupation, Education
- What could be the way forward in the prevention and control of NCD at community level?
- Is there anything else that you would like to tell us so that we have a better understanding of the NCD situation in the community, or are there any issues you feel are important that we have not touched on during the interview?

At the end of the discussion:

- Thank the focus group for taking time to share the valuable information
